# Analysis of Time-Series Gene Expression Data to Explore Mechanisms of Chemical-Induced Hepatic Steatosis Toxicity

**DOI:** 10.3389/fgene.2018.00396

**Published:** 2018-09-18

**Authors:** Alejandro Aguayo-Orozco, Frederic Yves Bois, Søren Brunak, Olivier Taboureau

**Affiliations:** ^1^Novo Nordisk Foundation Center for Protein Research, Faculty of Health and Medical Sciences, University of Copenhagen, Copenhagen, Denmark; ^2^Institut National de l’Environnement Industriel et des Risques (INERIS), Unité Modèles pour l’Ecotoxicologie et la Toxicologie (METO), Verneuil en Halatte, France; ^3^UMRS 973 INSERM, Université Paris Diderot, Université Sorbonne Paris Cité, Paris, France

**Keywords:** hepatic steatosis, gene expression, transcriptomics, time-series analysis, pathways analysis, drug induced liver injury, DILI

## Abstract

Non-alcoholic fatty liver disease (NAFLD) represents a wide spectrum of disease, ranging from simple fatty liver through steatosis with inflammation and necrosis to cirrhosis. One of the most challenging problems in biomedical research and within the chemical industry is to understand the underlying mechanisms of complex disease, and complex adverse outcome pathways (AOPs). Based on a set of 28 steatotic chemicals with gene expression data measured on primary hepatocytes at three times (2, 8, and 24 h) and three doses (low, medium, and high), we identified genes and pathways, defined as molecular initiating events (MIEs) and key events (KEs) of steatosis using a combination of a time series and pathway analyses. Among the genes deregulated by these compounds, the study highlighted OSBPL9, ALDH7A1, MYADM, SLC51B, PRDX6, GPAT3, TMEM135, DLGDA5, BCO2, APO10LA, TSPAN6, NEURL1B, and DUSP1. Furthermore, pathway analysis indicated deregulation of pathways related to lipid accumulation, such as fat digestion and absorption, linoleic and linolenic acid metabolism, calcium signaling pathway, fatty acid metabolism, peroxisome, retinol metabolism, and steroid metabolic pathways in a time dependent manner. Such transcription profile analysis can help in the understanding of the steatosis evolution over time generated by chemical exposure.

## Introduction

Non-alcoholic fatty liver disease (NAFLD) is diagnosed increasingly worldwide and is considered to be the most common liver disorder in the West (Rector et al., 2008). NAFLD refers to a spectrum of hepatic disorders, ranging from simple hepatic steatosis with no apparent specific symptoms to hepatocellular carcinoma (Jozefczuk et al., 2012). Hepatic steatosis is caused by abnormal accumulation of triglycerides (TG) in the liver due to chemical exposures other than excessive alcohol consumption. This accumulation of TG in vesicles impairs hepatic function and makes the liver highly susceptible to other injuries related to metabolic syndrome and systemic energy metabolism (Marchesini et al., 2003). Simultaneously, it affects the local immune system and that may lead to more severe autoimmune diseases (Antherieu et al., 2011). From a metabolic point of view, steatosis occurs when the fatty acids (FAs) influx or synthesis in the liver exceeds the capacity to clear them. The metabolic pathways leading to the development of hepatic steatosis are multiple, including enhanced non-esterified FA release from adipose tissue (lipolysis), increased *de novo* FAs (lipogenesis) and decreased β-oxidation (Fuchs et al., 2014).

Some toxicogenomics studies have been reported on drug-induced steatosis (DIS) or steatohepatitis (DISH) (Lake et al., 2011; Starmann et al., 2012; Hebels et al., 2014; Rabinowich and Shibolet, 2015), however, the mechanisms of action leading to steatosis are not fully understood. To support toxicity evidence with mechanistic pathways and mode of action for drug safety and risk assessment, the OECD has recently developed the adverse outcome pathway (AOP) concept. The AOP concept involves all the essential steps that take place in the toxicity pathways, from the molecular initiating event (MIE) at the protein or gene level, passing through organelle effect, cellular, tissue, organ and finally population effect. One key principle is that AOPs are chemical agnostic pathways (Vinken, 2013). Steatosis is one of the AOPs highly investigated and although AOPs are chemical agnostic pathways, the activation of some specific molecules, which lead to the over or under regulation of key events (KEs) with steatosis as a final outcome has been reported on the OECD AOP website^1^.

In this study, we decided to analyze transcriptomic data on a set of 28 drugs tested in primary human hepatocytes and suspected to cause steatosis. In order to obtain an overall understanding of the disease, steatosis-producing chemicals were compiled and analyzed together. An interesting feature is that compounds have been studied at different times and doses, so we were able to perform a time-series analysis at the gene level but also at the pathway level. The distinction between time points and concentrations can explain how the different KEs affect one another, which will help in explaining complex hepatotoxicity. The results of our analysis support previously reported finding and provide new hypotheses that could be investigated further.

## Materials and Methods

### Chemicals

For the current analysis, 28 compounds were selected according to their ability to induce steatosis in primary human hepatocytes (PHH) and the availability of gene expression data in the TG-GATEs (Toxicogenomics Project–Genomics-Assisted Toxicity Evaluation System) database (Igarashi et al., 2015). Furthermore, seven non-steatotic compounds available in TG-GATEs were also included according to the study carried out by Sahini and Borlak (2014) and Sahini et al. (2014) as negative controls. The negative controls have been associated with other histopathological observations in rat *in vivo* such as necrosis, cellular infiltration, fibrosis and granuloma (**Supplementary Table S1**). The TG-GATEs database contains data from PHH exposed to those compounds and collected using Affymetrix HG U133 Plus 2.0 gene expression microarrays. Two replicates were tested at three dose levels (low, medium, and high) and at three time points (2, 8, and 24 h after initial dosing). For each experiment, corresponding untreated controls are also tested. The 35 chemicals used in the study are summarized in **Table 1**. The specific dose and times can be found in **Supplementary Table S2**.

**Table 1:** Compounds used in the analysis.

Compound name	Abbreviation	Cas No.	Sample	Reference
Allyl Alcohol	AA	107-18-6	Steatosis	(Waterfield et al., 1993)
Amiodarone	AM	1951-25-3	Steatosis	(Antherieu et al., 2011)
Acetaminophen	APAP	103-90-2	Steatosis	(Fontana, 2008)
Acetamide	AAA	60-35-5	Steatosis	(Zhang et al., 2017)
Amitriptyline	AMT	50-48-6	Steatosis	(Xia et al., 2000)
Aspirin	ASA	50-78-2	Steatosis	(Shen et al., 2014)
Coumarin	CMA	91-64-5	Steatosis	(Sahini et al., 2014)
Colchicine	COL	64-86-8	Steatosis	(Seiliez et al., 2016)
Clomipramine	CPM	303-49-1	Steatosis	(Xia et al., 2000)
Cyclosporin A	CSA	59865-13-3	Steatosis	(Lopez-Riera et al., 2017)
Clozapine	CZP	5786-21-0	Steatosis	(Zhang et al., 2007)
Diltiazem	DIL	42399-41-7	Steatosis	(Dowman et al., 2010)
Disulfiram	DSF	97-77-8	Steatosis	(Balakirev and Zimmer, 2001)
Ethanol	ETN	64-17-5	Steatosis	(Donohue, 2007)
Ethinylestradiol	EE	57-63-6	Steatosis	(Morii et al., 2014)
Ethionamide	ETH	536-33-4	Steatosis	(Zhang et al., 2017)
Hydroxyzine	HYZ	68-88-2	Steatosis	(Sahini et al., 2014)
Imipramine	IMI	50-49-7	Steatosis	(Xia et al., 2000)
Lomustine	LS	13010-47-4	Steatosis	(King and Perry, 2001)
Methapyrilene	MP	91-80-5	Steatosis	(Craig et al., 2006)
Methyltestosterone	MTS	58-18-4	Steatosis	(Schoonen et al., 2007)
Phenylbutazone	PhB	50-33-9	Steatosis	(Bessone, 2010)
Rifampicin	RIF	13292-46-1	Steatosis	(Tostmann et al., 2008)
Terbinafine	TBF	91161-71-6	Steatosis	(Choudhary et al., 2014)
Tetracycline	TC	60-54-8	Steatosis	(Antherieu et al., 2011)
Vitamin A	VA	68-26-8	Steatosis	(Liu et al., 2016)
Valproic acid	VPA	99-66-1	Steatosis	(Vitins et al., 2014)
Pirinixic acid	WY	50892-23-4	Steatosis	(Cannon and Eacho, 1991)
Carbamazepine	CBZ	298-46-4	Negative Control	(Bessone, 2010)
Diclofenac	DFNa	15307-86-5	Negative Control	(Bessone, 2010)
Indomethacin	IM	53-86-1	Negative Control	(Dehpour et al., 1999)
Naproxen	NP	22204-53-1	Negative Control	(Bessone, 2010)
Nifedipine	NIF	21829-25-4	Negative Control	(Basile and Mascia, 1999)
Nimesulide	NIM	51803-78-2	Negative Control	(Bessone, 2010)
Sulindac	SUL	103-90-2	Negative Control	(Bessone, 2010)

*The reference corresponds to the publication in which drug-induced hepatic steatosis or negative control has been reported.*

### Microarray Data Analysis

All data were analyzed using the robust multi-array average (RMA) methodology in the Bioconductor R package for background-adjusted, normalized, and log-transformed perfect matched values of individual probes from the Affymetrix Human Genome U133 Plus 2.0 array (Irizarry et al., 2003). 54,675 probes corresponding to 19,945 uniquely annotated Gene Symbol IDs define each microarray. There is a total of 225 experiments according to concentration, time of exposure and compound used for the treatment. These experiments where analyzed in four steps: (1) all the experiments have been normalized concertedly. Such global normalization highlights the most important genes, which are those affected by the toxicity of more than one compound, and most likely in more than one time point and/or concentration (Krug et al., 2013). When dealing with gene expression microarray data, results can be affected by small differences in any number of non-biological variables, i.e., reagents or different technicians. (2) The two replicates per compound and condition were averaged. (3) Batch effect was accounted in the design matrix, reducing the bias effect on further steps of the analysis similarly to what has been performed by Grimberg et al. (2014). Concretely, for each gene a linear model following Eq. 1 (corresponding to a *t*-test comparison between two groups) was performed (Ritchie et al., 2015). (4) Subsequently, differentially expressed genes (DEGs) were calculated by dividing the average signal obtained from the chemical exposed group by the average signal from control receiving the vehicle only. The Student *t*-test was used to calculate the *p* value which was corrected by Bonferroni multiple testing. Finally, DEGs were selected by considering the *p* values less than 0.05 and fold-changes higher than 1.5. Genes that met these criteria also in the negative control set were removed from the deregulated gene’s list for steatosis, assuming that these genes were not related to steatosis.

(1) $$Y_{\mathrm{ij}}=\alpha_{j}+x_{i}\beta_{j}+\varepsilon_{\mathrm{ij}}$$

### Time-Series Analysis

To characterize the deregulation of genes related to steatosis over time, after drug administration, a time-series analysis was performed on the 28 compounds using the package MasigPro in R (Nueda et al., 2014). This analysis was performed for each compound individually. With MasigPro, genes with significant temporal expression changes were selected and their variance at the different concentration (low, medium, and high) were analyzed. As a first step, a regression on time for each gene taking all the variables present in the model, hence using all the genes, was performed. A false discovery rate (FDR) method was used to select genes with a value less than 0.05. Moreover, for each gene the best regression model was selected using stepwise regression. A backward method was used; therefore all genes were used as variables to initialize the modeling (*p*-value <0.05 were considered). In a final step, the R-squared of the regression model was used as cut-off value in order to reduce the amount of false positive findings (genes). R-squared was set to 0.6 to allow flexibility to the regression model, since we are working with all the compounds associated with steatosis, as suggested by MasigPro. Overall, MasigPro provides information on genes that change over time and in respect to the control. Such analysis can be visualized, plotting DEG of every single gene for each compound studied according to time and dose.

### Gene Set and Pathway Analysis

In addition to the DEG and the time-series analysis, a pathway analysis was performed based on our gene expression analysis for the 28 compounds. Compared with the individual gene/molecule-based approach, pathway analysis is more sensitive, consistent and informative on the outcomes studied (Luo et al., 2009). In our study, the parametric statistical analysis model (PAGE) was used (Kim and Volsky, 2005). The method is based on a modified Gene Set Enrichment Analysis (GSEA). A gene randomization test was applied to the gene expression data, in which the significance of gene sets is identified for pathways (computing permutations of gene labels or a parametric distribution over genes). The database used for the study of the pathways was KEGG, which is a database resource that integrates genomic, chemical and systemic functional information for a large set of pathways (Kanehisa et al., 2012). In order to obtain a quantitative result of the compound’s effect over the pathways, a Gene Fold Enrichment (GFE) score was calculated. This score divided the number of genes deregulated by the total number of genes of the pathway being analyzed and then multiply by the statistical mean for the same pathway. Pathview, a tool set for pathway-based data integration and visualization, was used within the GAGE package in R for visualization of the genes deregulated in the KEGG pathway (Luo and Brouwer, 2013). In our context, no specific pathway has been developed for steatosis in KEGG or other pathway databases. So, we have considered the NAFLD pathway, which is the closest pathway to steatosis for the visualization.

### Clustering

Previous studies have shown that the use of gene expression clustering can group samples in clusters that may lead to a good prediction of the gene-outcome relationship (Alizadeh et al., 2000; Handen and Ganapathiraju, 2015). Therefore, a pathway analysis was also performed on the set of compounds after clustering. Clustering was based on the logarithm base 2 of the fold change of the gene expression at the different conditions. The Euclidian distance implemented in Ward.D2 in R method was used. The clustering method was performed in all compounds containing information for at least 2 timepoints, hence excluding clozapine (CZP) from the analysis, which has been studied only for 1 timepoint. The clustering has been performed separately for the different time points. The clustering shown in **Figure 5** was performed on the compounds at 24 h. The determination of the number of clusters was done by the *elbow method*. A range of *k* values from 1 to 10, *k* value being the number of chemical belonging to a cluster, were considered in our analysis. For each *k* value the sum of squared errors (SERs) was calculated and the selection of the number of cluster was based on a compromise between the number of clusters and low SER. *K* = 4 was selected as it showed a close to maximum separation of the samples and low SER.

## Results

### DEG Analysis

Firstly, we analyzed DEGs under all different conditions versus control for the 28 compounds with a global normalization of the 255 experiments (all together analysis). 742 genes are highly deregulated in at least one condition, i.e., one compound at a specific time and concentration (logFC ≥ 1.5 and with a FDR Bonferroni-corrected value ≤0.05).

**FIGURE 1:**
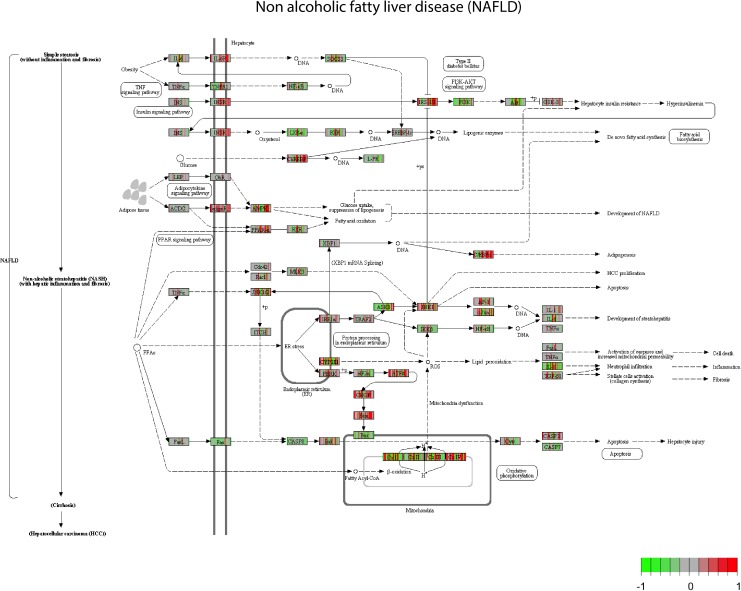
Non-alcoholic fatty liver disease (NAFLD) pathway view. After a GAGE analysis of the gene expression data amongst the highest scored pathways (*p* < 0.05). All the compounds suspected to produce steatosis at middle concentration and time 8 h are plotted. All the gene expressions have been normalized from –1 to 1, centered at 0. Green boxes correspond to a down regulation of the genes by a chemical and red box an up-regulation of the genes.

For the pathway analysis, we looked specifically at the NAFLD pathway, a general pathway related to fatty liver, and for which steatosis might be related for some genes. Through the pathway enrichment analysis, many genes involved in the NAFLD are deregulated (**Figure 1**). Mapping the genes deregulated by the set of 28 compounds on the NAFLD pathway led to the observation that some genes are up regulated, in red (INSR, adipR, or PPARα), by a large set of compounds (AAA, PhB, CPM, and HYZ), whereas another set of genes is more often down regulated, in green (LXR, PI3K, FAS, CASP8, IKKB, and BAX) by others compounds (VA, ASA, and APAP). There are several compounds that show opposite effects by up/down-regulating the same genes. This is the case of CYP2E, AMPK and other mitochondrial genes. This supposes that there are different mechanisms of action that can trigger steatosis.

**FIGURE 2:**
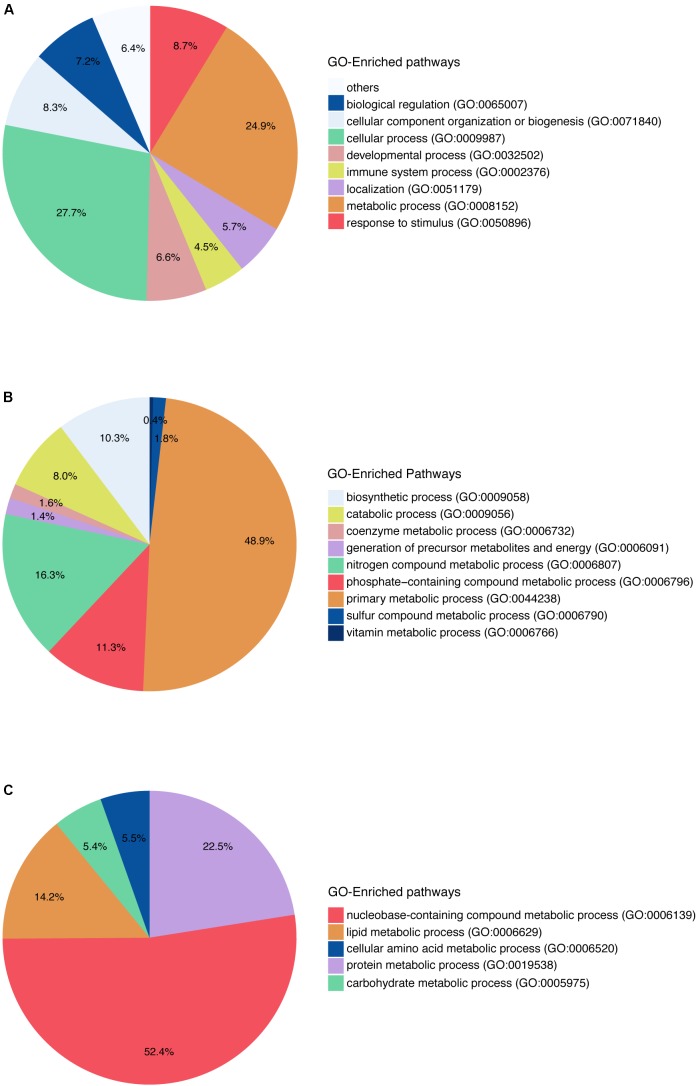
Gene Ontology pathway enrichment for *in vitro* human hepatocytes. **(A)** The two main blocks of pathways that are deregulated according to the genes that have a log2FC above absolute value 1.5 and a *q*-value ≤0.05, are affecting the metabolic pathways as well as the cellular processes in general. **(B)** The pathways affected within the metabolic pathways are shown here, and they affect mainly the primary metabolic process. **(C)** Pathways represented within primary metabolic process.

Gene Ontology pathway enrichment was also performed with the 742 genes in order to get an impression of the biological processes that were affected (**Figure 2**). The enrichment in terms of pathways based on GO terms was used in this study. At the first level of the pathway hierarchy, the deregulated genes are related to several pathways, including cellular process, metabolic process, localization, developmental process and immune system process. The two most significant are the cellular processes and metabolic processes. Within metabolic processes, primary metabolic processes are the most significant pathways targeted by the deregulated genes. In primary metabolic processes, the two most targeted pathways are nucleobase-containing compound metabolic processes and lipid metabolic processes. The latest contains steroid metabolic process, phospholipids metabolic process and FA metabolic process. Looking into the specific pathways, the most represented in the GO analysis are FA β-oxidation and acetyl-CoA metabolic process. So, we can note that many genes deregulated by the set of compounds affect lipids and FAs and play a role in steatosis.

### Time-Series Analysis

In the previous analysis, the outcomes were analyzed independently of time and dose. To investigate the evolution of the expression over time, a time-series analysis was carried out using the R package MasigPro. After removing the genes involved in cell cycle according to GO biological processes (see **Supplementary Table S3**) (Barron and Li, 2016), MasigPro detected 48 genes with significant temporal expression changes (**Table 2**). These genes are mainly involved in metabolic and immune system pathways. Among them, some genes have previously been reported to play a hepatotoxic role such as MYADM (Megger et al., 2014), SLC51B (Arab et al., 2017), PRDX6 ((Newton et al., 2009; Pacifici et al., 2014), OSBPL9 (Hong and Tontonoz, 2014), GPAT3 (Khatun et al., 2016), TMEM135 (Exil et al., 2010), DLGDA5 (Liao et al., 2013), BCO2 (Ip et al., 2015), IDH3G (Pan et al., 2014), NEURL1B (Lawan et al., 2015), and TSPAN6 (Wang et al., 2012). An extensive work done in rodents related to steatosis adverse outcome described how OSBPL proteins promote the development of NAFLD in mice (Stein et al., 2017). Finally, the role of GPTA proteins has been reported to play a role in the development of hepatic steatosis (Yu et al., 2018).

**Table 2:** Deregulated genes over time and dose.

Entrez-ID	Gene symbol	Gene full name
91663	MYADM	Myeloid associated differentiation marker
27286	SRPX2	Sushi repeat containing protein, X-linked 2
10491	CRTAP	Cartilage associated protein
84803	GPAT3	Glycerol-3-phosphate acyltransferase 3
9787	DLGAP5	DLG associated protein 5
83875	BCO2	Beta-carotene oxygenase 2
3161	HMMR	Hyaluronan mediated motility receptor
3421	IDH3G	Isocitrate dehydrogenase 3 (NAD(+)) gamma
6790	AURKA	Aurora kinase A
28998	MRPL13	Mitochondrial ribosomal protein L13
54492	NEURL1B	Neuralized E3 Ubiquitin Protein Ligase 1B
2633	GBP1	Guanylate binding protein 1
10112	KIF20A	Kinesin family member 20A
80114	BICC1	BicC family RNA binding protein 1
65084	TMEM135	Transmembrane protein 135
7105	TSPAN6	Tetraspanin 6
9615	GDA	Guanine deaminase
9488	PIGB	Phosphatidylinositol glycan anchor biosynthesis class B
55771	PRR11	Proline rich 11
11167	FSTL1	Follistatin like 1
2519	FUCA2	Fucosidase, alpha-L-2, plasma
9588	PRDX6	Peroxiredoxin 6
79594	MUL1	Mitochondrial E3 ubiquitin protein ligase 1
51292	GMPR2	Guanosine monophosphate reductase 2
81610	FAM83D	Family with sequence similarity 83 member D
55872	PBK	PDZ binding kinase
59	ACTA2	Actin, alpha 2, smooth muscle, aorta
7802	DNALI1	Dynein axonemal light intermediate chain 1
5445	PON2	Paraoxonase 2
3242	HPD	4-hydroxyphenylpyruvate dioxygenase
28998	MRPL13	Mitochondrial ribosomal protein L13
11004	KIF2C	Kinesin family member 2C
1606	DGKA	Diacylglycerol kinase alpha
10158	PDZK1IP1	PDZK1 interacting protein 1
9122	SLC16A4	Solute carrier family 16 member 4
23082	PPRC1	Peroxisome proliferator-activated receptor gamma, coactivator-related 1
123264	SLC51B	Solute carrier family 51 beta subunit
6372	CXCL6	C-X-C motif chemokine ligand 6
79053	ALG8	ALG8, alpha-1,3-glucosyltransferase
9928	KIF14	Kinesin family member 14
788	SLC25A20	Solute carrier family 25 member 20
114883	OSBPL9	Oxysterol binding protein like 9
55526	DHTKD1	Dehydrogenase E1 and transketolase domain containing 1
56922	MCCC1	Methylcrotonyl-CoA carboxylase 1
10351	ABCA8	ATP binding cassette subfamily A member 8
501	ALDH7A1	Aldehyde dehydrogenase 7 family member A1
516	ATP5G	ATP Synthase Membrane Subunit C Locus 1
9488	PIGB	Phosphatidylinositol glycan anchor biosynthesis class B

Our results confirm previous transcriptomics analysis in rodents with deregulation of genes such as GPAT, KIF, CXCL, and SLC family genes (Sahini et al., 2014) and OSBP family that alters the lipid metabolism in mice (Béaslas et al., 2013).

**FIGURE 3:**
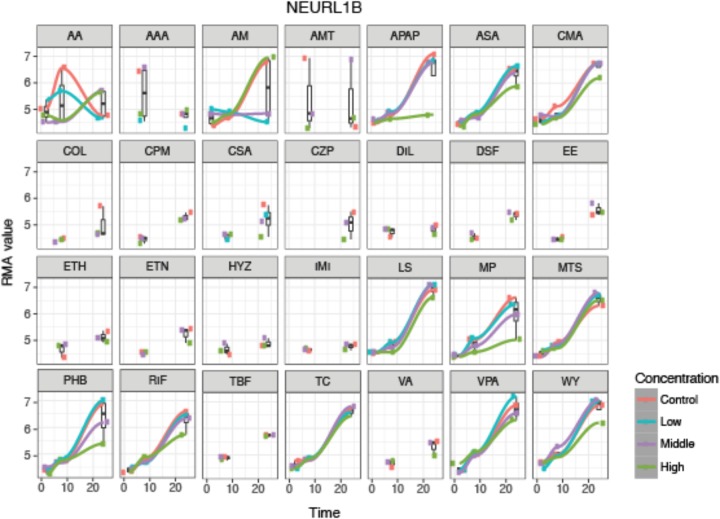
De-regulation over time of the 28 steatotic chemicals for the selected protein, NEURL1B, from the time-series analysis. The *y*-axis indicates the normalized expression value of the gene at every time-point, 2, 8 and 24 h (*x*-axis), where the different colors indicate the dosages (red indicates control, blue low dose, purple middle dose, and green high dose). It shows a time dependency of the protein expression and compound specific dose effect. However, it does not show a systematic change on the gene expression due to dose. For some compounds only two time-point samples were taken.

An example of the visualization of the time-series analysis is shown in **Figure 3** for neutralized E3 ubiquitin protein ligase 1B (NEURL1B) after exposure to the 28 compounds. We can observe that NEURL1B is regulated in positive direction over time for many compounds. Other examples are presented in supplementary information (**Supplementary Figure S1**).

From the gene list, other genes might play a role in steatosis and could be investigated further. For example, ALDH7A1 is highly deregulated in a time/dose-dependent manner. This gene is involved in oxidoreductase mechanisms and in protection of cell against oxidative stress by metabolizing a number of lipid peroxidation-derived aldehydes and could lead to steatosis. For some compounds, i.e., AM, APAP, LS, and MP, the de-regulation of ALDH7A1 is also dependent on the treatment concentration. The deregulation of these proteins will lead to a higher production of lipids, which together with a reduction of beta-oxidation of lipids could promote their accumulation in cells. Finally, a set of compounds deregulated some genes differently, suggesting that they trigger steatosis through another mechanism. This is the case for example for DSF and EE, which showed a weak deregulation of OSBL9 and a higher deregulation of the ALDH7A1.

Some genes known to be commonly associated to steatosis in human are not in this top list. This is the case for example of PNPLA3, which does not appear as one of the highest deregulated genes in our study. The genetic variation in PNPLA3 has been previously shown to play a role in the increase of FA accumulation in liver leading to steatosis (Romeo et al., 2008). So, the lack of the specific polymorphism related to susceptibility to steatosis in the cells used could explain the non-deregulation of this gene in our study.

Overall, this list of genes provides an insight into the mechanistic pathways already related to steatosis, as well as new hypotheses that can be analyzed further. Interestingly, the expression for many genes vary a little from control as a function of dose and the difference in the pattern of expression between control and treatment is relatively low. This confirmed a previous analysis showing that the doses differences between treatments in rat primary hepatocytes explain less than 0.1% of variation in all cases (Sutherland et al., 2016). One possible explanation is primary hepatocytes rapidly dedifferentiate (Lauschke et al., 2016) which could generate a gradual down regulation of hepatocyte function over time in culture.

### Pathway Time-Series Analysis

**FIGURE 4:**
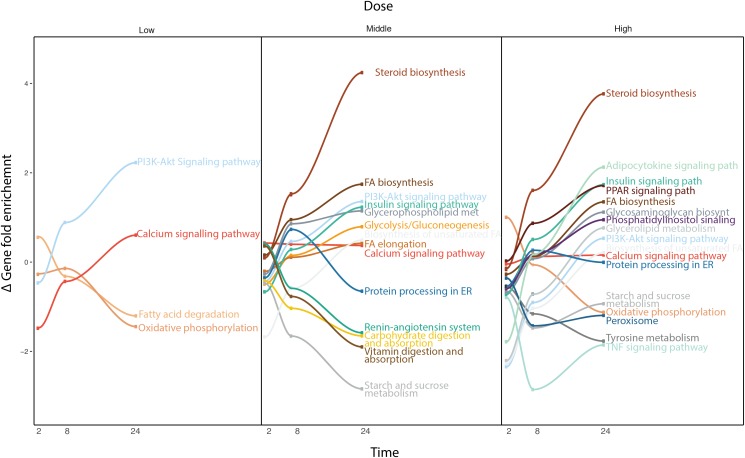
Time-series pathway analysis based on GFE and depending on the concentration. The *y*-axis displays the overall Gene Fold Enrichment.

Due to the broad pharmacological and physicochemical characteristics of the compounds used for this study, we developed a new type of time-series analysis at the pathway level. For this purpose, all the compounds were analyzed to obtain the most significantly deregulated pathways including their corresponding GFE score (**Figure 4**).

At low concentration, pathways such as FA degradation and oxidative phosphorylation start to get down regulated. The deregulation of the oxidative phosphorylation is prominently affecting the mitochondria, therefore reducing the activity within this organelle, such as β-oxidation, which is the catabolic process through which FAs are broken down. The PIK3-Akt signaling pathway gets up regulated through the activation of the AMPK signaling pathway or downstream, Mtor signaling pathway, affecting the metabolism of the cell (Li et al., 2010). At higher concentrations, other important pathways are affected. The steroid biosynthesis is up-regulate at middle and high dose. FA biosynthesis and FA elongation are also among the up regulated pathways. Hence more FAs and lipids are produced. In contrast, the protein processing in ER, known to be related to lipid homeostasis, is down regulated. Interestingly, several studies have previously shown the existence of comorbidities between liver diseases and cardiovascular (CDV) diseases (Anstee et al., 2018). The deregulation of the renin-angiotensin system could explain part of the relation between steatosis and any possible CDV disease. Vitamin digestion and absorption is down regulated, which also points toward de-regulation in the FA β-oxidation. Also tyrosine metabolism, which is related to liver damage displays down-regulation. When the liver is damaged, phenylalanine cannot be converted to tyrosine. At this highest concentration, the adipocytokine signaling pathway and TNFα signaling pathway are deregulated, which indicates an activation of the cellular immune system. This immune system de-regulation may contribute the steatotic condition to move forward to other more severe drug-induced liver damages. Note that at high dose, cells often develop non-specific toxicity and the pathways altered may be not related solely to steatosis but also to other toxicity endpoints. The pathway analysis confirmed the little contribution of doses over time at the gene level observed previously, as the majority of the pathways deregulated in middle dose are also present in high dose.

**FIGURE 5:**
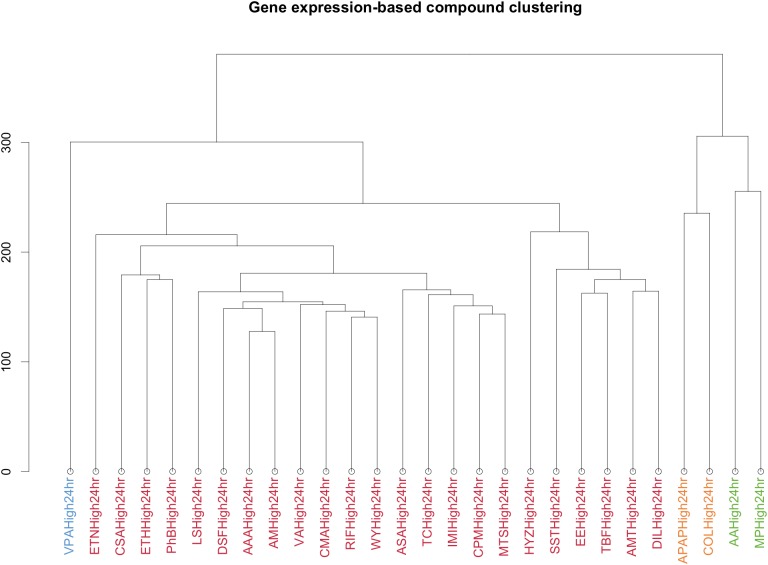
Clustering by Euclidean distance of the different compounds based on the logarithm in base 2 Fold Change of the gene expression for the different conditions. Each color represents a cluster. The *y*-axis is a measure of closeness of either cluster or individual data points.

Finally, to obtain a more characteristic view on the specific action points of the different compounds, we performed a similar analysis after clustering the compounds through the gene’s signature similarity. Using the Euclidean distance based on the log2FC of the gene expression, all compounds were clustered in four sets (**Figure 5**).

VPA was clustered separately. MP and AA formed a different cluster as well as APAP and COL. A final cluster contained the remaining compounds. This last cluster contains essentially drugs used to treat a variety of conditions, acting as immunosuppressant’s, antineoplastic agents, antibiotics, biguanides and butylpyrazolidines.

**FIGURE 6:**
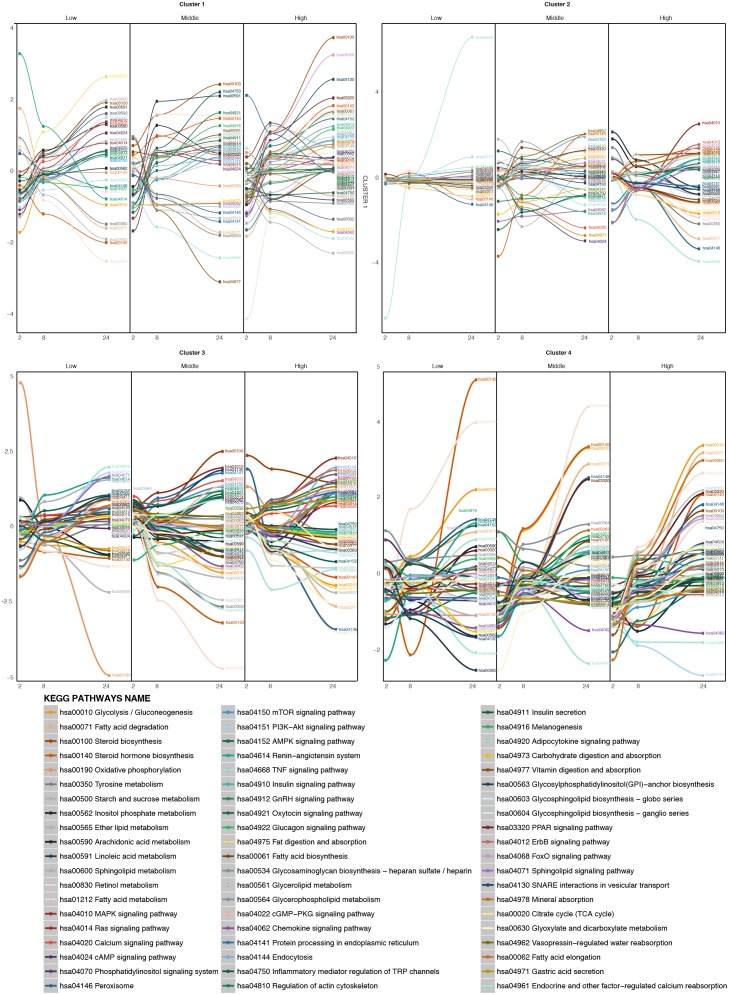
Time-series pathway analysis based on GFE and depending on the concentration for the four clustered of compounds. The *y*-axis displays the overall Gene Fold Enrichment.

After clustering, the pathway time-series analysis was performed on each of the four clusters (**Figure 6**). For the compounds of the larger cluster, cluster 1 (TBF, AMT, DIL, PhB, HYZ, CSA, WY, RIF, ASA, AM, AAA, EE, VA, TC, MTS, IMI, CPM, DSF, CMA, ETH, LS, ETN), at low concentrations of treatment metabolic pathways such as fat digestion and absorption, linoleic and linolenic acid metabolism, calcium signaling pathway and others are up regulated over time. Other pathways, such as FA metabolism, peroxisome, retinol metabolism, and some steroid metabolic pathways are down regulated. At higher concentrations from time 2 to 24 h, the FA metabolism, calcium signaling pathway and steroid hormone biosynthesis increase over time, showing a de-regulation of these pathways promoted by the treatment. The FA degradation pathway is down regulated. It means that the FAs inside the cell are increasing and there are no pathways to deplete them. The oxidative phosphorylation becomes down regulated over time. So, the oxidative conditions in the mitochondria are starting to be reduced at this concentration. Finally, at the highest concentration tested, many signaling pathways known to be steatosis-producing related are targeted. FoxO, MAPK, PPAR signaling pathways, are highly up regulated. Steroid hormone biosynthesis, FA biosynthesis, glycerophospholipid metabolism, glycosphyngolipid biosynthesis and other lipid metabolic pathways are also up regulated. Moreover, SNARE interactions in vesicular transport are also up regulated over time, which could indicate the internalization of the FAs into vesicles, and so accumulation inside the cells. In contrast, pathways, such as oxidative phosphorylation, vitamin digestions and absorption and FA degradation are down regulated over time.

For cluster 2 (AA, MP) (**Figure 6**) at low concentrations the most highly up regulated pathways are the PI3K-Akt signaling pathway, the Mtor signaling pathway and the adipocytokine signaling pathway. Some metabolic pathways like FA degradation, peroxisome and retinol metabolism are down regulated. With the increasing concentration, steroid biosynthesis starts to be up regulated. At the highest concentration, glycolysis/gluconeogenesis, FA degradation, TNFα signaling pathway, tyrosine metabolism, peroxisome, PPAR signaling pathway and retinol metabolism become down-regulated over the time and FA metabolism, adipocytokine signaling pathway, MAPK signaling pathway, among others, become up-regulated. This could be explained by a lesser effect of these compounds. Therefore higher concentrations are needed in order to deregulate the cell to a steatotic pattern.

In the case of cluster 3 (APAP, COL) (**Figure 6**), at low concentrations, oxidative phosphorylation is highly down regulated, together with retinol metabolism and some lipid metabolism such as ether lipid metabolism and glycolysis/gluconeogenesis. On the other hand, FA elongation, TNFα signaling pathway, PI3K-Akt signaling pathway and sphingolipid signaling pathway and sphingolipid metabolism are highly up regulated. At higher concentrations, retinol metabolism continues to be down-regulated, glycolysis/gluconeogenesis, FA elongation are down-regulated and protein processing in the ER, steroid biosynthesis, SNARE interactions in vesicular transport among others are up regulated. These deregulations could affect the export of FAs to the exterior of the cell and their accumulation within organelles.

For the last cluster, containing only VPA (**Figure 6**) at low, middle and high doses, FA degradation, PPAR signaling pathway and retinol metabolism, all of them involved in the elimination of FAs are up regulated. This compound produces a strong effect on the metabolism of FAs and lipids and therefore the cells react with increasing the pathway activities associated with FA degradation.

So, it is interesting to see that, each of the cluster shows some pathway deregulation related lipid metabolism, FA degradation, glycolysis or PPAR signaling pathway, all related to steatosis.

## Discussion

The conventional assumption that a drug acts selectively on a single target is shifting toward “drug-holistic” systems based approaches. Similarly, a disease or a toxicity endpoint reflects not only the impairment of a unique gene. In fact, the disruption of many genes and pathways can lead to a disease or a specific toxicity. In the case of steatosis, we have focused the study on trying to understand the underlying mechanisms for steatosis using a set of diverse compounds. Considering the MIEs and KEs known to lead to the AOP steatosis (based on AOP-Wiki), our study confirms the deregulation of these biomarkers and highlighted new genes that produce steatosis. With the development of a time-series analysis combined with pathway analysis, it is possible to follow the evolution of the pathways over time and how they are connected to the different stages of steatosis.

Interestingly, the integration of a large and diverse set of compounds in the analysis pinpoints their specificity in leading to steatosis. However, our results show that the time seems to have a higher impact in the DEGs and pathways analysis than the concentration. The early dedifferentiation of PHH in 2D cultures might explain this observation. It is also possible that the global normalization reduces the specific signal of some genes. Additionally, for more than half of the compounds studied, only two times points have been tested experimentally, which might influence the results.

In our study, the compounds have been tested in PHH and the translation to human liver tissue would be of great interest to validate these outcomes. Some rats *in vivo* data beyond the 24 h time point are available in TG-GATEs and could be analyzed similarly in order to evaluate the overlap between *in vitro* and *in vivo* data. Finally, it has been reported that 3D cell cultures could be a more suitable system to mimic human organs that 2D cultures (Fey and Wrzesinski, 2012) and it would be interesting to assess the steatogenic effect of these compounds in this 3D spheroid system.

**FIGURE 7:**
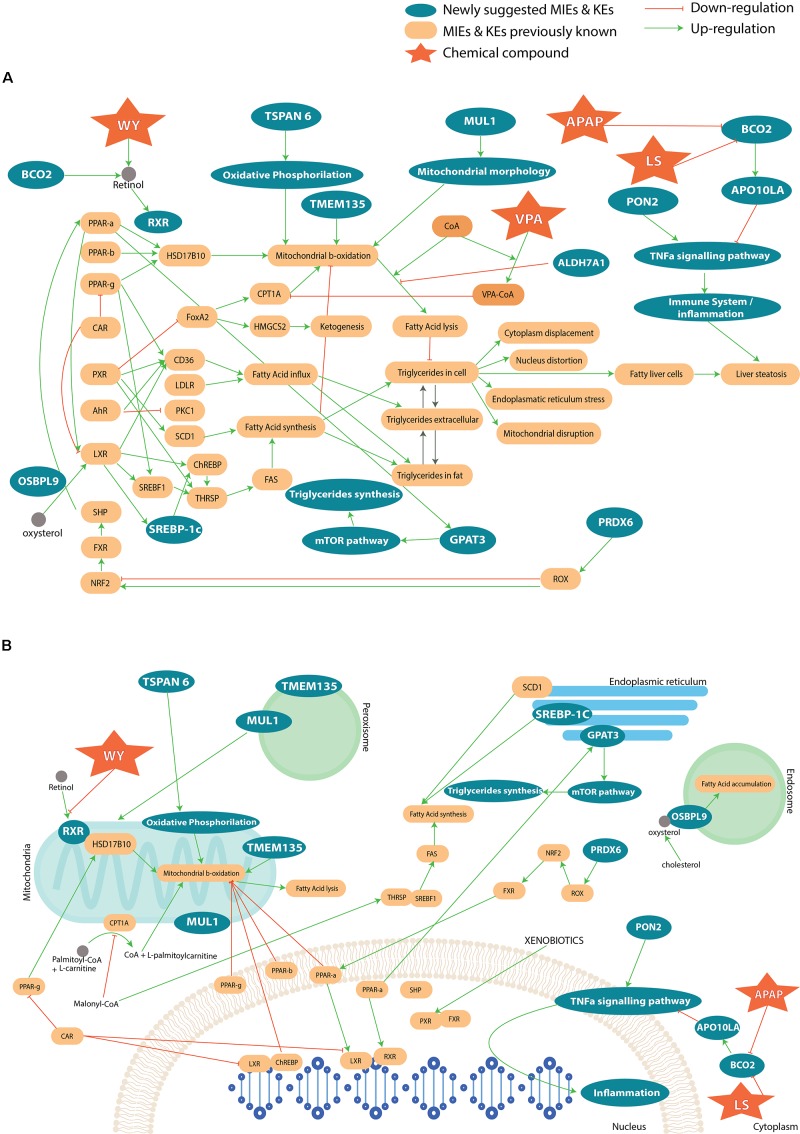
**(A)** Adverse Outcome Pathway related to steatosis network, which summarizes a collection of information across several AOPs. The yellow boxes represent known genes and key events associated to steatosis. The blue color shows the newly suggested genes and pathways involved in the AOPs. Only compounds to which there is a higher likelihood to affect the MIE or KE than other compounds have been introduced in the AOP. In **(B)** the different cell compartments and how the interact with each other in the pathways that lead to steatosis.

To summarize, most of the genes that are associated with a steatosis AOP, described in AOP wiki, have been found in our study. The integration of the previously published information on steatosis with the newly found genes and pathways from our analysis can enrich the knowledge of developed AOPs on steatosis. The **Figure 7A** represents an AOP network, i.e., the result of an accumulation of a number of individual AOPs listed on the AOP Wiki website. The list of AOPs used for the completion of the full steatosis pathway is: 34 (LXR activation leading to hepatic steatosis), 36 (Peroxisomal Fatty Acid Beta-Oxidation Inhibition Leading to Steatosis), 57 (AhR activation leading to hepatic steatosis), 58 (NR1I3 (CAR) suppression leading to hepatic steatosis), 60 (NR1I2 (Pregnane X Receptor, PXR) activation leading to hepatic steatosis), 61 (NFE2L2/FXR activation leading to hepatic steatosis). Besides, the capture of coenzyme A by VPA was added to the mechanistic pathway (Schumacher and Guo, 2015), as well as oxidative stress (Spahis et al., 2017). In this figure, we can see direct (and indirect) interaction between genes suggested by the analysis and known genes. For example, TSPAN6 deregulates oxidative phosphorylation, which acts on the mitochondrial β-oxidation. The deregulation of ALDH7A1 will lead to a higher production of lipids and impact the oxidative stress with a reduction of β-oxidation. In contrast, PON2 impacts the immune system. We looked also at the cellular compartmental level and how the genes deregulation can perturb the interaction with each other and lead to steatosis (**Figure 7B**). We can see that all the cell compartments can be involved in steatosis, many of which undertake functions within the mitochondria and the nucleus. More specifically, perturbation in endoplasmic reticulum and vesicles through the genes MUL1, TMEM135, OSBPL9, SCD1, SREBP-1C, GPAT3 can lead to steatosis.

Other studies have reported computational approaches to leverage large-scale toxicogenomic information, biological pathways and high throughput data for the identification of toxicity pathways. For example, Bell et al. (2016) described a computational approach in which curated biological pathways and high-throughput toxicity data are used to identify toxicity pathways. This computational method uses a data-driven approach to assemble an AOP, which allows for the integration of biological information into pathway-based networks and can be updated with new information. Coupling both approaches could be interesting in the enrichment of the steatosis AOPs.

Overall, our findings illustrate how an integrative computational chemical system biology approach can be used to study steatosis and obtain new metabolic pathways that are deregulated during the process of liver injury by chemical exposure. Obviously, these findings need to be further validated with additional experimental studies. These associations are potentially not causative but more reflect biomarkers along the pathway to develop steatosis. In many case, changes in gene expression are a response to a stressor and it is only when these adaptive changes are overwhelmed that the adverse effect occurs.

## Author Contributions

AA-O performed the experiments. AA-O and OT analyzed the results and wrote the paper. FB and SB contributed in the writing of the final manuscript.

## Conflict of Interest Statement

The authors declare that the research was conducted in the absence of any commercial or financial relationships that could be construed as a potential conflict of interest.

## Footnotes

1 https://aopwiki.org/

## Supplementary Material

FIGURE S1 Time-series analysis for a set gene involved in steatosis.

TABLE S1 Histopathology findings in rat *in vivo* assays for the negative control compounds. The compounds are listed together with the dose level and time at which the pathological finding happened.

TABLE S2 List of compounds used in the analysis with the specific time points, the exact dose and the dose level.

TABLE S3 Genes removed from results table after time-series analysis for the role in cell cycle pathways. Filter carried out by selecting genes that appear in any cell cycle related pathway from GO Biological processes.
