# Supplementary material for: Analysis of Time-Series Gene Expression Data to Explore Mechanisms of Chemical-Induced Hepatic Steatosis Toxicity
Source: Front Genet. 2018 Sep 18;9:396. doi: 10.3389/fgene.2018.00396 (PMC6153316; doi:10.3389/fgene.2018.00396)
Supplement: TABLE S1 — Histopathology findings in rat in vivo assays for the negative control compounds. The compounds are listed together with the dose level and time at which the pathological finding happened. [file Data_Sheet_1.PDF]

Compound	Dose Level	Time	Pathology finding type
carbamazepine	Low	6 hr	Cellular infiltration
carbamazepine	Low	6 hr	Necrosis
carbamazepine	Low	15 day	Hypertrophy
carbamazepine	Low	29 day	Cellular infiltration
carbamazepine	Low	29 day	Hypertrophy
carbamazepine	Middle	8 day	Necrosis
carbamazepine	Middle	15 day	Hypertrophy
carbamazepine	Middle	15 day	Necrosis
carbamazepine	Middle	29 day	Hypertrophy
carbamazepine	High	6 hr	Cellular infiltration
carbamazepine	High	24 hr	Cellular infiltration
carbamazepine	High	4 day	Cellular infiltration
carbamazepine	High	8 day	Hypertrophy
carbamazepine	High	15 day	Granuloma
carbamazepine	High	15 day	Hypertrophy
carbamazepine	High	29 day	Hypertrophy
diclofenac	Low	8 day	Cellular infiltration
diclofenac	Low	8 day	Necrosis
diclofenac	Low	15 day	Cellular infiltration
diclofenac	Middle	4 day	Necrosis
diclofenac	Middle	4 day	Nodule, hepatodiaphragmatic
diclofenac	Middle	8 day	Necrosis
diclofenac	Middle	29 day	Cellular infiltration
diclofenac	High	8 day	Cellular infiltration
diclofenac	High	8 day	Scar
diclofenac	High	15 day	Cellular infiltration
indomethacin	Low	9 hr	Necrosis
indomethacin	High	24 hr	Nodule, hepatodiaphragmatic
indomethacin	High	8 day	Change, basophilic
indomethacin	High	8 day	DEAD
indomethacin	High	8 day	Inflammation, suppurative
indomethacin	High	15 day	Change, basophilic
indomethacin	High	15 day	DEAD
indomethacin	High	15 day	Inflammation, suppurative
indomethacin	High	15 day	Necrosis
naproxen	High	24 hr	Hematopoiesis, extramedullary
naproxen	High	4 day	Hematopoiesis, extramedullary
naproxen	High	4 day	Hypertrophy
naproxen	High	4 day	Inflammation
naproxen	High	4 day	Necrosis

naproxen	High	8 day	DEAD
naproxen	High	8 day	Hematopoiesis, extramedullary
naproxen	High	15 day	DEAD
naproxen	High	15 day	Hematopoiesis, extramedullary
naproxen	High	15 day	
nifedipine	Middle	3 hr	Increased mitosis
nifedipine	Middle	6 hr	Increased mitosis
nifedipine	Middle	29 day	Change, eosinophilic
nifedipine	High	3 hr	Increased mitosis
nifedipine	High	6 hr	Increased mitosis
nifedipine	High	9 hr	Increased mitosis
nifedipine	High	15 day	Change, eosinophilic
nifedipine	High	29 day	Change, eosinophilic
nimesulide	Low	3 hr	Microgranuloma
nimesulide	Low	6 hr	Microgranuloma
nimesulide	Low	9 hr	Microgranuloma
nimesulide	Low	24 hr	Increased mitosis
nimesulide	Low	24 hr	Microgranuloma
nimesulide	Low	4 day	Microgranuloma
nimesulide	Low	8 day	Microgranuloma
nimesulide	Low	15 day	Microgranuloma
nimesulide	Low	15 day	Necrosis
nimesulide	Low	29 day	Fibrosis
nimesulide	Low	29 day	Microgranuloma
nimesulide	Middle	6 hr	Microgranuloma
nimesulide	Middle	4 day	Microgranuloma
nimesulide	Middle	8 day	Hypertrophy
nimesulide	Middle	8 day	Microgranuloma
nimesulide	Middle	15 day	Hypertrophy
nimesulide	Middle	15 day	Microgranuloma
nimesulide	Middle	15 day	Necrosis
nimesulide	Middle	29 day	Ground glass appearance
nimesulide	Middle	29 day	Hypertrophy
nimesulide	Middle	29 day	Microgranuloma
nimesulide	Middle	29 day	Necrosis
nimesulide	High	3 hr	Increased mitosis
nimesulide	High	6 hr	Microgranuloma
nimesulide	High	9 hr	DEAD
nimesulide	High	4 day	Hypertrophy
nimesulide	High	8 day	DEAD
nimesulide	High	8 day	Ground glass appearance
nimesulide	High	8 day	Hypertrophy
nimesulide	High	8 day	Microgranuloma
nimesulide	High	8 day	Necrosis

nimesulide	High	15 day	Ground glass appearance
nimesulide	High	15 day	Hypertrophy
nimesulide	High	29 day	Ground glass appearance
nimesulide	High	29 day	Hypertrophy
nimesulide	High	29 day	Microgranuloma
sulindac	High	24 hr	Hypertrophy
sulindac	High	8 day	Atrophy
sulindac	High	8 day	Cellular infiltration, neutrophil
sulindac	High	15 day	DEAD
sulindac	High	15 day	Hematopoiesis, extramedullary
sulindac	High	29 day	DEAD
sulindac	High	29 day	Hematopoiesis, extramedullary
sulindac	High	29 day	Hypertrophy
