# Supplementary material for: Analysis of Time-Series Gene Expression Data to Explore Mechanisms of Chemical-Induced Hepatic Steatosis Toxicity
Source: Front Genet. 2018 Sep 18;9:396. doi: 10.3389/fgene.2018.00396 (PMC6153316; doi:10.3389/fgene.2018.00396)
Supplement: TABLE S2 — List of compounds used in the analysis with the specific time points, the exact dose and the dose level. [file Data_Sheet_2.PDF]

Compound	Dose (μM)	Dose Level	Time (hours)
Allyl Alcohol	2,80	Low	2
Allyl Alcohol	2,80	Low	8
Allyl Alcohol	2,80	Low	24
Allyl Alcohol	14,00	Middle	2
Allyl Alcohol	14,00	Middle	8
Allyl Alcohol	14,00	Middle	24
Allyl Alcohol	70,00	High	2
Allyl Alcohol	70,00	High	8
Allyl Alcohol	70,00	High	24
Amiodarone	0,28	Low	2
Amiodarone	0,28	Low	8
Amiodarone	0,28	Low	24
Amiodarone	1,40	Middle	2
Amiodarone	1,40	Middle	8
Amiodarone	1,40	Middle	24
Amiodarone	7,00	High	2
Amiodarone	7,00	High	8
Amiodarone	7,00	High	24
Acetaminophen	200,00	Low	2
Acetaminophen	200,00	Low	8
Acetaminophen	200,00	Low	24
Acetaminophen	1000,00	Middle	2
Acetaminophen	1000,00	Middle	8
Acetaminophen	1000,00	Middle	24
Acetaminophen	5000,00	High	2
Acetaminophen	5000,00	High	8
Acetaminophen	5000,00	High	24
Acetamide	400,00	Low	8
Acetamide	400,00	Low	24
Acetamide	2000,00	Middle	8
Acetamide	2000,00	Middle	24
Acetamide	10000,00	High	8
Acetamide	10000,00	High	24
Aspirin	120,00	Low	2
Aspirin	120,00	Low	8
Aspirin	120,00	Low	24
Aspirin	600,00	Middle	2
Aspirin	600,00	Middle	8
Aspirin	600,00	Middle	24
Aspirin	3000,00	High	2
Aspirin	3000,00	High	8
Aspirin	3000,00	High	24
Coumarin	12,00	Low	2
Coumarin	12,00	Low	8
Coumarin	12,00	Low	24
Coumarin	60,00	Middle	2
Coumarin	60,00	Middle	8

Coumarin	60,00	Middle	24
Coumarin	300,00	High	2
Coumarin	300,00	High	8
Coumarin	300,00	High	24
Colchicine	800,00	Middle	8
Colchicine	800,00	Middle	24
Colchicine	4000,00	High	8
Colchicine	4000,00	High	24
Clomipramine	2,00	Middle	8
Clomipramine	2,00	Middle	24
Clomipramine	10,00	High	8
Clomipramine	10,00	High	24
Cyclosporin A	0,24	Low	8
Cyclosporin A	0,24	Low	24
Cyclosporin A	1,20	Middle	8
Cyclosporin A	1,20	Middle	24
Cyclosporin A	6,00	High	8
Cyclosporin A	6,00	High	24
Clozapine	10,00	Middle	24
Clozapine	50,00	High	24
Diltiazem	30,00	Middle	8
Diltiazem	30,00	Middle	24
Diltiazem	150,00	High	8
Diltiazem	150,00	High	24
Dilsulfiram	12,00	Middle	8
Dilsulfiram	12,00	Middle	24
Dilsulfiram	60,00	High	8
Dilsulfiram	60,00	High	24
Ethanol	2000,00	Middle	8
Ethanol	2000,00	Middle	24
Ethanol	10000,00	High	8
Ethanol	10000,00	High	24
Ethinylstradiol	3,00	Middle	8
Ethinylstradiol	3,00	Middle	24
Ethinylstradiol	15,00	High	8
Ethinylstradiol	15,00	High	24
Ethionamide	120,00	Middle	8
Ethionamide	120,00	Middle	24
Ethionamide	600,00	High	8
Ethionamide	600,00	High	24
Hydroxizine	8,00	Middle	8
Hydroxizine	8,00	Middle	24
Hydroxizine	40,00	High	8
Hydroxizine	40,00	High	24
Imipramine	3,00	Middle	8
Imipramine	3,00	Middle	24
Imipramine	15,00	High	8
Imipramine	15,00	High	24

Lomustine	4,80	Low	2
Lomustine	4,80	Low	8
Lomustine	4,80	Low	24
Lomustine	24,00	Middle	2
Lomustine	24,00	Middle	8
Lomustine	24,00	Middle	24
Lomustine	120,00	High	2
Lomustine	120,00	High	8
Lomustine	120,00	High	24
Methapyrilene	24,00	Low	2
Methapyrilene	24,00	Low	8
Methapyrilene	24,00	Low	24
Methapyrilene	120,00	Middle	2
Methapyrilene	120,00	Middle	8
Methapyrilene	120,00	Middle	24
Methapyrilene	600,00	High	2
Methapyrilene	600,00	High	8
Methapyrilene	600,00	High	24
Methyltestosterone	0,80	Low	2
Methyltestosterone	0,8	Low	8
Methyltestosterone	0,8	Low	24
Methyltestosterone	4,0	Middle	2
Methyltestosterone	4,0	Middle	8
Methyltestosterone	4,0	Middle	24
Methyltestosterone	20,0	High	2
Methyltestosterone	20,0	High	8
Methyltestosterone	20,0	High	24
Phenylbutazone	16,0	Low	2
Phenylbutazone	16,0	Low	8
Phenylbutazone	16,0	Low	24
Phenylbutazone	80,0	Middle	2
Phenylbutazone	80,0	Middle	8
Phenylbutazone	80,0	Middle	24
Phenylbutazone	400,0	High	2
Phenylbutazone	400,0	High	8
Phenylbutazone	400,0	High	24
Rifampicin	2,8	Low	2
Rifampicin	2,8	Low	8
Rifampicin	2,8	Low	24
Rifampicin	14,0	Middle	2
Rifampicin	14,0	Middle	8
Rifampicin	14,0	Middle	24
Rifampicin	70,0	High	2
Rifampicin	70,0	High	8
Rifampicin	70,0	High	24
Terbinafine	3,0	Middle	8
Terbinafine	3,0	Middle	24
Terbinafine	15,0	High	8

Terbinafine	15,0	High	24
Tetracycline	1,0	Low	2
Tetracycline	1,0	Low	8
Tetracycline	1,0	Low	24
Tetracycline	5,0	Middle	2
Tetracycline	5,0	Middle	8
Tetracycline	5,0	Middle	24
Tetracycline	25,0	High	2
Tetracycline	25,0	High	8
Tetracycline	25,0	High	24
Vitamin A	1,5	Middle	8
Vitamin A	1,5	Middle	24
Vitamin A	7,5	High	8
Vitamin A	7,5	High	24
Valproic acid	200,0	Low	2
Valproic acid	200,0	Low	8
Valproic acid	200,0	Low	24
Valproic acid	1000,0	Middle	2
Valproic acid	1000,0	Middle	8
Valproic acid	1000,0	Middle	24
Valproic acid	5000,0	High	2
Valproic acid	5000,0	High	8
Valproic acid	5000,0	High	24
Pirinixic acid	6,0	Low	2
Pirinixic acid	6,0	Low	8
Pirinixic acid	6,0	Low	24
Pirinixic acid	30,0	Middle	2
Pirinixic acid	30,0	Middle	8
Pirinixic acid	30,0	Middle	24
Pirinixic acid	150,0	High	2
Pirinixic acid	150,0	High	8
Pirinixic acid	150,0	High	24
Carbamazepine	12,0	Low	2
Carbamazepine	12,0	Low	8
Carbamazepine	12,0	Low	24
Carbamazepine	60,0	Middle	2
Carbamazepine	60,0	Middle	8
Carbamazepine	60,0	Middle	24
Carbamazepine	300,0	High	2
Carbamazepine	300,0	High	8
Carbamazepine	300,0	High	24
Diclofenac	16,0	Low	2
Diclofenac	16,0	Low	8
Diclofenac	16,0	Low	24
Diclofenac	80,0	Middle	2
Diclofenac	80,0	Middle	8
Diclofenac	80,0	Middle	24
Diclofenac	400,0	High	2

Diclofenac	400,0	High	8
Diclofenac	400,0	High	24
Indomethacin	8,0	Low	2
Indomethacin	8,0	Low	8
Indomethacin	8,0	Low	24
Indomethacin	40,0	Middle	2
Indomethacin	40,0	Middle	8
Indomethacin	40,0	Middle	24
Indomethacin	200,0	High	2
Indomethacin	200,0	High	8
Indomethacin	200,0	High	24
Naproxen	120,0	Middle	8
Naproxen	120,0	Middle	24
Naproxen	600,0	High	8
Naproxen	600,0	High	24
Nifedipine	30,0	Middle	8
Nifedipine	30,0	Middle	24
Nifedipine	150,0	High	8
Nifedipine	150,0	High	24
Nimesulide	66,0	Middle	8
Nimesulide	66,0	Middle	24
Nimesulide	330,0	High	8
Nimesulide	330,0	High	24
Sulindac	600,0	Middle	8
Sulindac	600,0	Middle	24
Sulindac	3000,0	High	8
Sulindac	3000,0	High	24
