# Supplementary material for: Analysis of Time-Series Gene Expression Data to Explore Mechanisms of Chemical-Induced Hepatic Steatosis Toxicity
Source: Front Genet. 2018 Sep 18;9:396. doi: 10.3389/fgene.2018.00396 (PMC6153316; doi:10.3389/fgene.2018.00396)
Supplement: TABLE S3 — Genes removed from results table after time-series analysis for the role in cell cycle pathways. Filter carried out by selecting genes that appear in any cell cycle related pathway from GO Biological processes. [file Data_Sheet_3.PDF]

Gene Symbol	Gene full name
DDB2	damage specific DNA binding protein 2(DDB2)
NEK2	NIMA related kinase 2(NEK2)
FOXM1	forkhead box M1(FOXM1)
PLK1	polo like kinase 1(PLK1)
HMOX1	heme oxygenase 1(HMOX1)
AXL	AXL receptor tyrosine kinase(AXL)
MGST2	microsomal glutathione S-transferase 2(MGST2)
TOR3A	torsin family 3 member A(TOR3A)
SNRNP25	small nuclear ribonucleoprotein U11/U12 subunit 25(SNRNP25)
HMGN1	high mobility group nucleosome binding domain 1(HMGN1)
KIF4A	kinesin family member 4A(KIF4A)
PAK1IP1	PAK1 interacting protein 1(PAK1IP1)
COQ2	coenzyme Q2, polyprenyltransferase(COQ2)
MR1	major histocompatibility complex, class I-related(MR1)
PTTG1	pituitary tumor-transforming 1(PTTG1)
COPS6	COP9 signalosome subunit 6(COPS6)
PSMD11	proteasome 26S subunit, non-ATPase 11(PSMD11)
CEBPZ	CCAAT/enhancer binding protein zeta(CEBPZ)
HIKESHI	Hikeshi, heat shock protein nuclear import factor(HIKESHI)
ASPA	aspartoacylase(ASPA)
KNL1	kinetochore scaffold 1(KNL1)
UPP1	uridine phosphorylase 1(UPP1)
CEP55	centrosomal protein 55(CEP55)
ISG20	interferon stimulated exonuclease gene 20(ISG20)
RPL7L1	ribosomal protein L7 like 1(RPL7L1)
PDZD11	PDZ domain containing 11(PDZD11)
SPAG5	sperm associated antigen 5(SPAG5)
LRRC47	leucine rich repeat containing 47(LRRC47)
PIGH	phosphatidylinositol glycan anchor biosynthesis class H(PIGH)
CDKN3	cyclin dependent kinase inhibitor 3(CDKN3)
KRT19	keratin 19(KRT19)
MDM2	MDM2 proto-oncogene(MDM2)
ACTR3	ARP3 actin related protein 3 homolog(ACTR3)

DEPDC1B	DEP domain containing 1B(DEPDC1B)
POLH	DNA polymerase eta(POLH)
BIRC5	baculoviral IAP repeat containing 5(BIRC5)
MAK16	MAK16 homolog(MAK16)
TNS1	tensin 1(TNS1)
RRM2B	ribonucleotide reductase regulatory TP53 inducible subunit M2B(RRM2B)
CDC20	cell division cycle 20(CDC20)
C16orf62	chromosome 16 open reading frame 62(C16orf62)
BYSL	bystin like(BYSL)
ALDH7A1	aldehyde dehydrogenase 7 family member A1(ALDH7A1)
EEPD1	endonuclease/exonuclease/phosphatase family domain containing 1(EEPD1)
COTL1	coactosin like F-actin binding protein 1(COTL1)
MKI67	marker of proliferation Ki-67(MKI67)
SUMF1	sulfatase modifying factor 1(SUMF1)
CENPF	centromere protein F(CENPF)
SPC25	SPC25, NDC80 kinetochore complex component(SPC25)
SPA17	sperm autoantigenic protein 17(SPA17)
TUSC1	tumor suppressor candidate 1(TUSC1)
GRWD1	glutamate rich WD repeat containing 1(GRWD1)
TOP2A	topoisomerase (DNA) II alpha(TOP2A)
CDCA3	cell division cycle associated 3(CDCA3)
